# Pan-Genomic and Transcriptomic Analyses of Marine *Pseudoalteromonas agarivorans* Hao 2018 Revealed Its Genomic and Metabolic Features

**DOI:** 10.3390/md20040248

**Published:** 2022-03-31

**Authors:** Yuhao Jv, Chenxiang Xi, Yanqiu Zhao, Wei Wang, Yiling Zhang, Kai Liu, Wenlin Liu, Kai Shan, Chunlei Wang, Ruiwen Cao, Cunxi Dai, Yiting Jv, Wenxing Zhu, Haiyong Wang, Qiuxia He, Lujiang Hao

**Affiliations:** 1 School of Bioengineering, Qilu University of Technology (Shandong Academy of Sciences), Jinan 250353, China; 10431201111@stu.qlu.edu.cn (Y.J.); 1043119587@stu.qlu.edu.cn (C.X.); 10431211120@stu.qlu.edu.cn (Y.Z.); 10431211258@stu.qlu.edu.cn (W.W.); 10431200980@stu.qlu.edu.cn (Y.Z.); kai_liu2018@163.com (K.L.); 1043118323@stu.qlu.edu.cn (W.L.); kai_shan@163.com (K.S.); chunlei_wang79@163.com (C.W.); rwcao@qlu.edu.cn (R.C.); 201996080002@stu.qlu.edu.cn (C.D.); 202181010040@stu.qlu.edu.cn (Y.J.); wenxingzhu@qlu.edu.cn (W.Z.); wangchemical@126.com (H.W.); heqiuxia2022@126.com (Q.H.); 2 State Key Laboratory of Biobased Material and Green Papermaking, Qilu University of Technology (Shandong Academy of Sciences), Jinan 250353, China

**Keywords:** *Pseudoalteromonas agarivorans*, pan-genome, genomic and metabolic features, KEGG analysis, transcriptome analysis

## Abstract

The genomic and carbohydrate metabolic features of *Pseudoalteromonas agarivorans* Hao 2018 (*P. agarivorans* Hao 2018) were investigated through pan-genomic and transcriptomic analyses, and key enzyme genes that may encode the process involved in its extracellular polysaccharide synthesis were screened. The pan-genome of the *P. agarivorans* strains consists of a core-genome containing 2331 genes, an accessory-genome containing 956 genes, and a unique-genome containing 1519 genes. Clusters of Orthologous Groups analyses showed that *P. agarivorans* harbors strain-specifically diverse metabolisms, probably representing high evolutionary genome changes. The Kyoto Encyclopedia of Genes and Genomes and reconstructed carbohydrate metabolic pathways displayed that *P. agarivorans* strains can utilize a variety of carbohydrates, such as d-glucose, d-fructose, and d-lactose. Analyses of differentially expressed genes showed that compared with the stationary phase (24 h), strain *P. agarivorans* Hao 2018 had upregulated expression of genes related to the synthesis of extracellular polysaccharides in the logarithmic growth phase (2 h), and that the expression of these genes affected extracellular polysaccharide transport, nucleotide sugar synthesis, and glycosyltransferase synthesis. This is the first investigation of the genomic and metabolic features of *P. agarivorans* through pan-genomic and transcriptomic analyses, and these intriguing discoveries provide the possibility to produce novel marine drug lead compounds with high biological activity.

## 1. Introduction

*Pseudoalteromonas agarivorans* is a gram-negative bacterium, obligate marine microorganism, and agar degrader that is motile, flagellated, and able to utilize organic matter as an energy source. The name *Pseudoalteromonas* was first proposed in 1995, following a reclassification from *Alteromonas* based on taxonomic analysis [1]. To date, researchers have isolated multiple strains of *P. agarivorans* from seawater samples around the globe [2,3]. The name *P. agarivorans* was first proposed in 2003 according to 16S ribosomal DNA sequencing analysis, DNA-DNA hybridization (DDH), and phylogenetic identification [3]. The type strain of this species is KMM 255^T^ (DSM 14585^T^).

Although few reports that characterize *P. agarivorans* are currently available, due to the specificity of the marine environment, they can secrete a variety of extracellular active substances, such as exopolysaccharides (EPS), antibiotics, and extracellular enzymes, which helps *P. agarivorans* to absorb nutrients and compete for survival [4,5]. Also, the *P. agarivorans* established its own complex marine niche using secreted EPS for antioxidant production [6], protection against low temperatures [7], flocculation promotion [8], and chelation of metal ions [9]. These strategies are not only effective for survival but also protective of the co-existing host species against adverse environmental conditions [10]. Komandrova et al. reported the structure of *O*-specific polysaccharides of *P. agarivorans* KMM232 (R type) [11] and KMM225^T^ [12] in 2010 and 2015, respectively. It was shown that the S-type and R-type marine bacteria KMM 232 synthesized *O*-specific lipopolysaccharides, which had different configurations compared to the extracellular polysaccharides (EPSs) secreted by the R-type and S-type terrestrial bacteria, and this variability reflects the adaptability of *P. agarivorans* to environmental changes. In the previous study, it was shown that the *P. agarivorans* Hao 2018 strain was a high-yield producer of EPS and had high biological activity in terms of anti-oxidation properties, moisture absorption and moisturization, adsorption of heavy metal ions, and in vitro flocculation [13,14]. Furthermore, the EPS produced by *P. agarivorans* Hao 2018 bacterium were methylated at low temperatures, which was beneficial for the expression of the glycosyltransferase gene in this bacterium [15,16]. In addition, we found that slight changes in environmental factors resulted in changes in the monosaccharide components and structure of the EPS produced by *P. agarivorans* Hao 2018, ultimately leading to changes in the biological activity of the EPS.

Using only one genome as a template for analyses does not provide complete genetic information about a species at the genetic level, especially when studying different subspecies or variants of the same species, in which the differences in unique genomes are typically more important than differences in core genomes. Therefore, the pan-genome approach is particularly significant. The pan-genome approach was proposed in 2005 by Tettelin et al. and has been applied in several fields because of its ability to interpret the genomic features of bacterial lineages as well as to analyze genome evolution. Pan-genome analysis with transcriptome analysis has helped to efficiently study the regulation of metabolic pathways and their applications [17,18,19,20]. Medigue et al. [21] first attempted genomic analysis of the genetic basis of *Pseudoalteromonas haloplanktis* TAC125, and when the solubility of oxygen increases at low temperatures, it responds by multiplying dioxygen scavenging while deleting the whole pathways that produce reactive oxygen species, thereby adapting to the low-temperature environment. Choudhury et al. [22] identified the *P. agarivorans* NW 4327 strain using 16S ribosomal RNA (rRNA), gyrase B gene sequences, DDH, and in silico genome comparisons. To the best of our knowledge, the genomic and carbohydrate metabolic features of *P. agarivorans* strains have been rarely explored, although they are widespread in the marine environment. Furthermore, John et al. [23] also used multiple bioinformatics approaches to identify a potential biosynthesis of AgNPs in a new *Marinomonas* strain isolated from the Antarctic psychrophilic ciliate *Euplotes focardii* that could be an alternative to conventional antibiotics. In this study, metabolic features were analyzed by phylogenetic and pan- genome analyses of *P. agarivorans*. The genomic profiles used were closely related to the *P. agarivorans* strains and were obtained from GenBank. We constructed the carbohydrate metabolic pathway of the bacteria by transcriptome analysis. In addition, we analyzed EPS synthesis at different nodes of time (pre-logarithmic growth phase and early stationary phase). In conclusion, this study helps to understand the phylogenetic diversity of *P. agarivorans* Hao 2018 and the regulation of its carbohydrate metabolic pathways.

## 2. Results

### 2.1. Genome Collection of the Pseudoalteromonas Strains

16S rRNA gene sequences of strain DSM 14585^T^, strain Hao 2018, and the closely related strains were used to build a phylogenetic tree (Figure S1). The criterion of 98.0% 16S rRNA gene sequence similarities to the two strains was used to collect all publicly available genomes putatively belonging to *P. agarivorans*. The genome sequences of all 10 strains used in this study were obtained from the GenBank database. The complete genome sequence of the *P. agarivorans* Hao 2018 strain, isolated from the microbial membrane on the surface of abalone seedlings, was obtained via whole-genome sequencing. We also obtained the complete genome physical map (GCA_003668795.1). At present, the NCBI database has complete genome sequences for two strains (*P. agarivorans* DSM 14585^T^ and *P. agarivorans* Hao 2018). The genome of *P. agarivorans* DSM 14585^T^ consisted of 2 chromosomes, whereas the *P. agarivorans* Hao 2018 genome consisted of a 0.12 Mb plasmid in addition to the 2 chromosomes [24].

**Figure 1:**
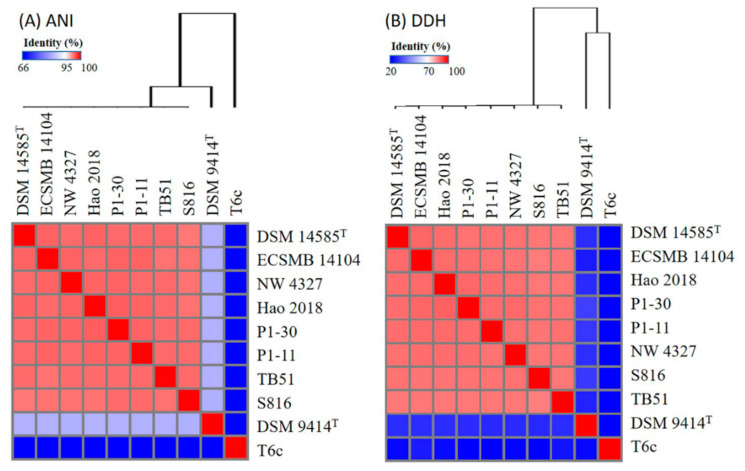
Heat maps comparing the ANI and DDH values of *P. agarivorans* Hao 2018 and other *Pseudoalteromonas* genomes.

The genome sequences of *P. agarivorans* DSM 14585^T^, *P. agarivorans* Hao 2018, *P. agarivorans* NW 4327, *P. agarivorans* S816, *P. atlantica* ECSMB14104, *P.* sp. P1–11, *P.* sp. P1–30, *P.* sp. TB51, *P. espejiana* DSM 9414^T^, and *P. atlantica* T6c were compared. The list of their general features is summarized in Table 1. Relatedness based on ANI (average nucleotide identity) and in silico DDH analyses were used to investigate the general features of *Pseudoalteromonas* genomes. According to the ANI analysis results (Figure 1), ANI values for *P. espejiana* DSM 9414^T^ and *P. atlantica* T6c were significantly lower than the classification thresholds used to distinguish between different species. The ANI values (97.8–98.3%) of all other strains were higher than the recommended ANI cutoff values (95–96%), indicating that they belonged to the same species [25,26,27]. Similarly, the results of the in silico DDH analysis showed that the DDH values of the DSM 9414^T^ and T6c strains were significantly lower compared to the in silico DDH values of all other strains (82–85.1%) as well as lower than the 70% DDH cutoff values. DDH values above the 70% threshold determined that the above two strains belonged to different species, while the remaining eight strains can be classified as *P. agarivorans* with the type strain DSM14585.

CheckM software was used to evaluate genome sequencing quality. The results are shown in Table 1. The completeness of all genome sequences was ≥91.0%, and the contamination was ≤4.0%. This was in line with the criteria for near-complete (≥90%) and low contamination (≤5%) [28]. As shown in the table, among the eight strains used for comparison, the genome of *P. atlantica* ECSMB14104 was the smallest (3.66 Mb) while the genome of *P.* sp. TB51 was the largest (4.63 Mb). The G + C content of these strains ranged from 40.8 to 41.0%. However, the genome of *P. agarivorans* NW 4327 and *P.* sp. TB51 contained numerous pseudogenes with frame shifts, most likely due to incomplete genes by numerous contigs or high rates of sequencing error, and was therefore excluded from the next pan-genome analysis of *Pseudoalteromonas* strains.

### 2.2. Pan- and Core-Genome Analyses of Pseudoalteromonas Strains

**Figure 2:**
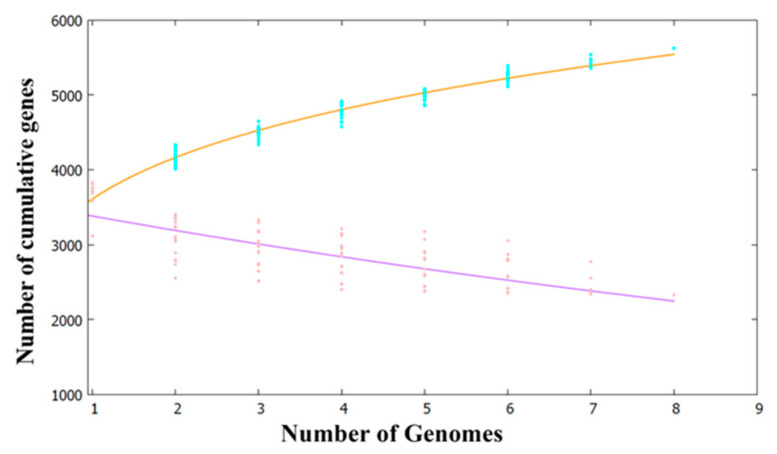
Pan-and core-genome analysis of eight *Pseudoalteromonas* strains. The orange and purple lines represent the pan-and core-genomes, respectively. The pan-genome represents the total number of genes in the genome in a sample subset, and the core-genome represents genes shared by all genomes in the same subset.

**Figure 3:**
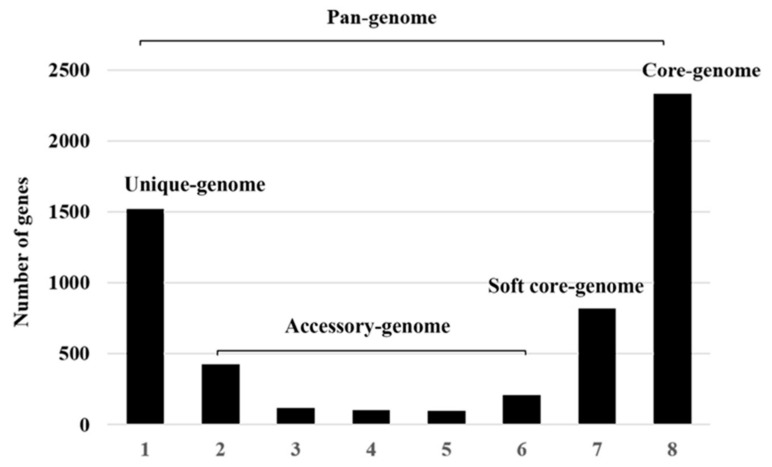
Histograms depicting the distribution of the pan-genome in *P. agarivorans*. ‘1’ represents the number of the unique genome, ‘2–6’ refers to the accessory genome, and ‘7’ and ‘8’ refers to the soft core-genome and the core genome, respectively.

A pan-genome analysis of the eight strains listed in Table 1 was performed using the BPGA pipeline and Heaps’ law (Figure 2). According to Heaps’ law, the size of the α exponent determines whether the bacterial pan-genome is in open or closed form. For example, if α < 1, the pan-genome is considered to be in open form, implying that adding more genomes will increase the size of the pan-genome. Conversely, for α > 1, the pan-genome is considered to be in closed form, implying that the addition of more genomic samples would not affect the size of the pan-genome. The genome analysis revealed that the pan-genomes of *Pseudoalteromonas* species increased with an α value of 0.20591, suggesting that may be an open pan-genome that has experienced frequent evolutionary changes and has been subject to effective environmental adaptation through gene gains and losses or lateral gene transfer. As shown in Figure 3, the pan-genome of the 8 *Pseudoalteromonas* strains contained a total of 5625 genes, including 2331 genes in the core-genome, 819 genes in the soft core-genome, 956 genes in the accessory genome, and 1519 genes in the unique genome. Unique genes that differ among *Pseudoalteromonas* strains, possibly reflect the different ecological niches (as residents of different geographic locations) and needs for the survival of *Pseudoalteromonas* strains [22].

### 2.3. Clusters of Orthologous Groups (COG) Analyses of P. agarivorans

**Figure 4:**
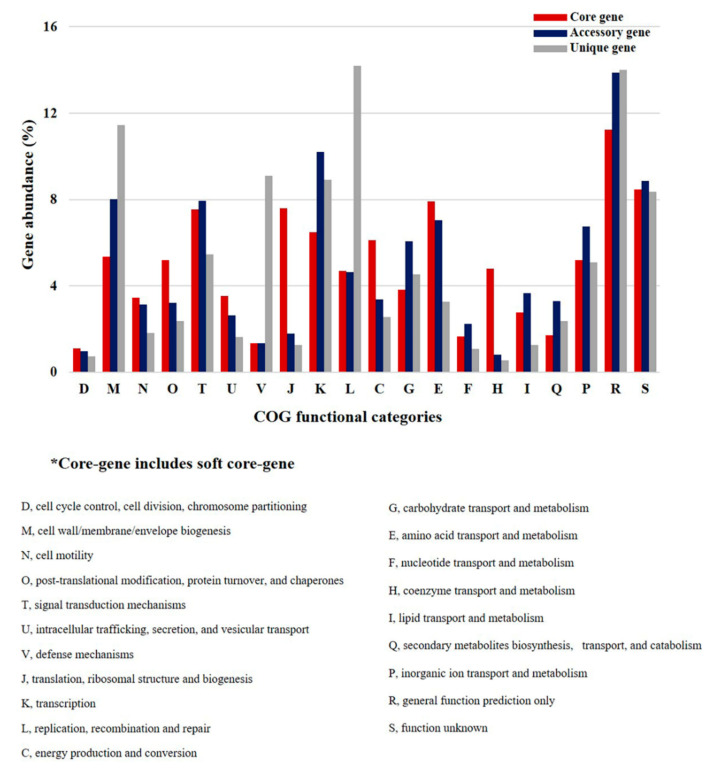
COG functional categories using the pan-genomes in *P. agarivorans* are shown.

*P. agarivorans* is a new type of marine bacterium, so a comparison with similar species could help predict its possible life activities. In order to study the metabolic and functional characteristics of *P. agarivorans*, we analyzed the COG distributions of the pan-genome and core-genome in this species (Figure 3). By annotating the core and accessory/unique genomes into specific COG functional categories, we were able to predict the possible functions of *P. agarivorans*. We observed some significant differences between the core- and accessory/unique genomes (Figure 4). We demonstrated its ability to produce EPS in previous studies [9]. As expected, genes involved in housekeeping processes, including genes responsible for cell cycle control, cell division, and chromosome partitioning (D); cell motility (N); post-translational modification, protein turnover, and chaperones (O); intracellular trafficking, secretion, and vesicular transport (U); translation, ribosomal structure, and biogenesis (J); energy production and conversion (C); amino acid transport and metabolism (E); and coenzyme transport and metabolism (H), had wide distribution in the core-genome compared to the accessory and unique genomes. The COG categories were mainly associated with energy metabolism or DNA repair, including cell wall/membrane/envelope biogenesis (M), defense mechanisms (V), transcription (K), carbohydrate transport and metabolism (G), and secondary metabolite biosynthesis, transport, and catabolism (Q) in the accessory and unique genes compared to the core genes. This indicated that the *P. agarivorans* strains possessed better carbohydrate fermentation properties, which might explain the high EPS yield.

### 2.4. KEGG and Carbohydrate Metabolism Regulation Analyses of P. agarivorans Hao 2018

**Figure 5:**
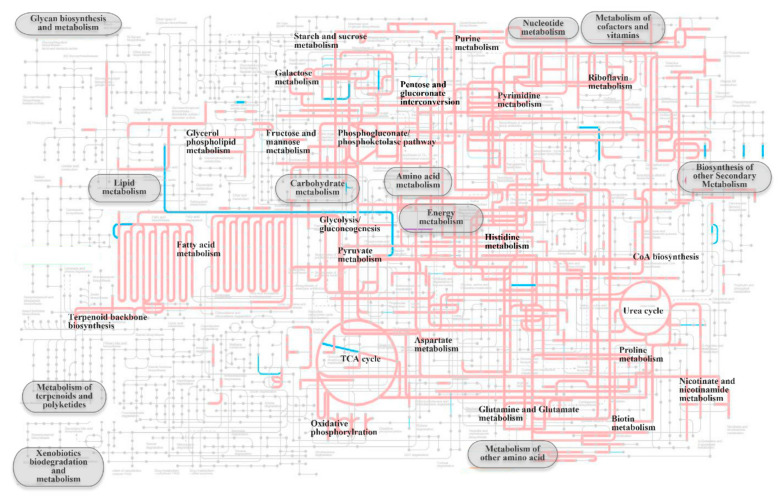
KEGG pathways of *P. agarivorans* Hao 2018. Line thickness is proportional to the number of genomes harboring the metabolic pathways. Metabolic pathways that were commonly present in *Pseudoalteromonas agarivorans* strains (7–8) are drawn in faint red, and non-common metabolic pathways in *Pseudoalteromonas agarivorans* strains (<7 strains) are depicted in sky blue.

**Figure 6:**
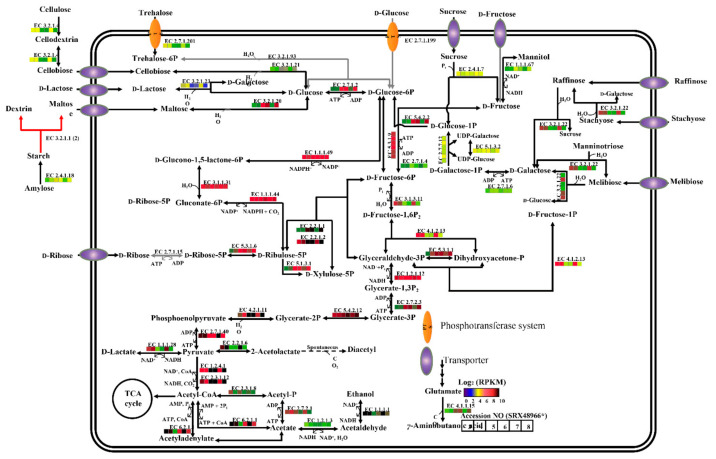
Carbohydrate metabolism regulation map of *P. agarivorans* Hao 2018. * The gene expression of enzymes related to carbohydrate metabolism in biosamples of *P. agarivorans* Hao 2018 were visualized by heatmaps based on their RPKM values.

In this study, the pan-genomic analysis shows higher gene abundance of unique genes than core and accessory genes encoding cell wall/membrane/envelope biogenesis in *Pseudoalteromonas* strains, which might be related to the fact that *Pseudoalteromonas* strains live in a special marine environment. Therefore, to further analyze key genes involved in cell wall/membrane/envelope biogenesis in *P. agarivorans* Hao 2018, we analyzed the KEGG and carbohydrate metabolism regulation of *P. agarivorans* Hao 2018. To investigate the metabolic characteristics and diversity of *Pseudoalteromonas agarivorans* Hao 2018, we mapped all functional genes of the seven *Pseudoalteromonas agarivorans* strains to their respective KEGG pathways (Figure 5). As shown in Figure 5, the KEGG pathway shows that the strains have highly similar metabolic and regulatory pathways, except for their marked differences in genome sizes and CDS numbers. The intracellular metabolism included starch and sucrose metabolism, galactose metabolism, fructose, and mannose metabolism, carbohydrate metabolism, fatty acid metabolism, energy metabolism, pyruvate metabolism, and amino acid metabolism pathways. Microbial growth is partially controlled by nutrient supply [29]. Gram-negative bacteria can enter and leave the stationary phase through complex physiological and morphological changes based on starvation [30]. The complete regulation of carbohydrate metabolism in the growth and metabolism of *P. agarivorans* is shown in Figure 6. We used transcriptomics to study the polysaccharide metabolism of *P. agarivorans* Hao 2018 at different growth stages. During the growth phase (from the beginning of logarithmic growth (2 h) to stable growth (24 h)) of *P. agarivorans* Hao 2018, using the genome as a reference background for the differential expression analysis of genes, we found that the expression of the gene encoding glucokinase was higher in the logarithmic growth phase (2 h) than in the stable phase (24 h). This led to a higher production of glucose-6-phosphate in cells, which could be used to synthesize EPS. Fructokinase gene expression was downregulated, indicating that the intracellular glucose and fructose conversion rates had decreased. At the same time, D-mannitol dehydrogenase gene expression was downregulated, and the conversion rate from fructose to mannose was reduced, which was consistent with the low mannose content of the EPS. The pyruvate pathway is linked to EPS production, and pyruvate carboxylase in the anaplerotic reaction provided intermediates for EPS synthesis [31,32]. Our study showed that pyruvate dehydrogenase in the 24 h culture was upregulated, which may have provided sufficient intermediates and/or energy for the synthesis of EPS. Interestingly, although the intracellular expression of genes involved in ribose and xylose synthesis was high, these sugars were not detected in EPS. Thus, intracellular xylose and ribose might have been used to synthesize other metabolites in cells.

### 2.5. Differentially Expressed Genes (DEGs) Analyses of P. agarivorans Hao 2018

**Figure 7:**
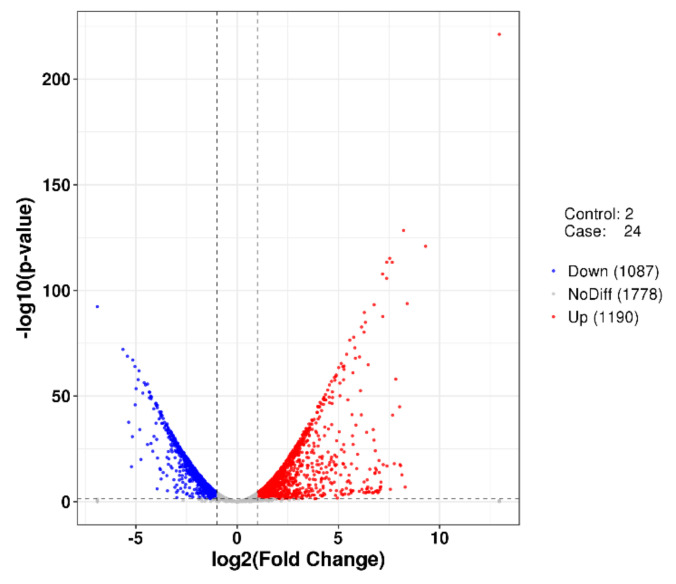
Volcano plot of differentially expressed genes between group 2 h and group 24 h. The vertical line is twice the expression difference threshold, and the horizontal line represents the *p*-value = 0.05. Red dots represent significantly upregulated genes and blue dots represent significantly downregulated genes (*p*-value < 0.05). Gray dots indicate non-significant differentially expressed genes.

Clean reads after quality control and filtering of raw data accounted for 99.65% of sequencing reads, with an average GC content of 43.65% (Table S1). Sequence mapping analysis showed that an average of 33,640,409 bp sequence could be mapped to the reference genome, accounting for 99.24% of the mapped sequence (Table S2). RPKM density distribution and statistical analysis of density distribution showed that moderately expressed genes accounted for the majority of the samples, with low and high expression genes accounting for a small proportion (Figure S2). The RPKM saturation of genes tends to saturate with sequencing results as the sampling proportion increases and the relative error decreases accordingly (Figure S3). The correlation test results of the samples showed that the correlation coefficients between the samples were all greater than 0.90, and the expression patterns between the samples were similar (Figure S4). In addition, the results of PCA analysis showed that the two groups of samples were significantly different in the first principal component (97% contribution), indicating that the samples were selected to be representative (Figure S5). The synthesis of bacterial EPS requires the participation of many types of genes, including transport-related genes, nucleotide sugar synthesis-related genes, and genes encoding glycosyltransferases. Analyses of DEGs showed that a total of 2277 genes were significantly differentially expressed in the two groups of samples, of which 1190 genes were up-regulated and 1087 genes were down-regulated in the stable phase compared to the logarithmic growth phase (Figure 7). Through Gene Ontology (GO) analysis and manual screening of the results of differentially expressed genes (DEGs) analysis, we obtained key genes that may encode enzymes related to exopolysaccharide synthesis. As shown in Table 2, the expression levels of genes encoding sugar transporters (D9T18_18920 and D9T18_05400) at 2 h were higher than those at 24 h. Monosaccharides need to be converted into corresponding nucleotide sugars before they can be used to synthesize EPS. Upon analysis of the expression of key enzyme genes in the process of nucleotide sugar synthesis (Table 3), it was found that the expression levels of genes encoding UDP-D-Glc (D9T18_02300, D9T18_06755, and D9T18_11960) at 2 h were higher than those at 24 h. This observation indicates that the strain can produce a large amount of glucose during the growth phase. Moreover, after the nucleotide sugar is synthesized, the main and side chains of the repeating unit are formed under the action of glycosyltransferases. A total of 15 genes encoding glycosyltransferase were found in the genome of *P. agarivorans* Hao 2018, and several of them showed differences in expression values between the 2 h and 24 h cultures (Table 4), suggesting that they may play different roles during EPS synthesis.

## 3. Discussion

Pan-genome and core-genome analyses are important methods for studying the genomic diversity and metabolic characteristics of microorganisms; they represent the genomic repositories of group members in a phylogenetic lineage [33,34]. The pan-and core-genomic analyses of *Pseudoalteromonas* found that it had an open genome, which indicates that it can adapt to environmental changes through gene expansion or reduction or horizontal gene transfer [35]. We noticed that the pan-genome of the eight *P. agarivorans* strains contained a total of 5625 genes, including 2331 genes in the core-genome and 1519 genes in the unique genome. The genes in the core-genome were responsible for the basic biological characteristics of major phenotypic traits while the unique genes differing among these strains reflected the different niches and survival needs of each strain [36,37]. This pan-genome and core-genome information could provide valuable evidence for studying the selective evolution of bacterial lineages under various environmental stressors. The genes in the core-genome were responsible for the basic cellular functions of *P. agarivorans*, whereas the accessory and unique genes represent the differential features and provide some survival advantages [38]. It is worth noting in the results of COG distribution analysis that the genes with replication, recombination, and repair (L) and defense mechanisms (V) and cell wall/membrane/envelope biogenesis (M) were over two times more enriched in the unique genes than in the core and accessory genes, which indicated that the strain has a good defense system against viruses or various pressures and can respond well to changes in the external environment; these results may explain the better antioxidant activity of the extracellular polysaccharides produced by *P. agarivorans* Hao 2018 at low temperature in our previous study [39]. Furthermore, the production of EPS may be an important strategy for marine bacteria to cope with external environmental perturbations. For example, *Idiomarina loihiensis* [40] can be attached to protein particles by synthesizing EPS, which improves its ability to use proteins and peptides to obtain the carbon and energy necessary for growth and development. The EPS produced by *A. macleodii* ‘deep ecotype’ [41] has a certain heavy metal binding capacity. So far, *Pseudoalteromonas* sp is one of the three main genera of marine EPS-producing bacteria that have been identified [42], our analysis revealed that a key gene encoding glucose-6-phosphate isomerase (Enzyme Commission (EC) 5.3.1.9) was identified from the genomes of *P. agarivorans* Hao 2018, which may confirm that the extracellular polysaccharide synthesis is one of the features of *P. agarivorans* Hao 2018 [9].

In addition, the analysis of differentially expressed genes showed that a total of 2277 genes were differentially expressed, of which 1189 genes were upregulated and 1088 genes were downregulated. When the strain *P. agarivorans* Hao 2018 was in the logarithmic growth phase (2 h), the expression of genes encoding glucokinase and phosphoglucomutase showed an upregulated trend, which may provide more glucose-1-phosphate for the synthesis of extracellular polysaccharides. Interestingly, the expression of the gene encoding rhamnosyl transferase was higher during the logarithmic growth period (2 h) than during the stable period (24 h), and whether this causes differences in the monosaccharide composition of extracellular polysaccharides between the two periods requires further verification. The results provide a new theoretical basis for further studying the metabolic regulation mechanism in response to changes in environmental factors. At the same time, we screened out some of the differentially expressed genes involved in the synthesis of EPS by analyzing the metabolic pathways of the bacterium, which provided inspiration for the targeted production of extracellular polysaccharides with good biological activity.

## 4. Materials and Methods

### 4.1. Strain Collection and Phylogenetic Analysis

Strain Hao 2018 was isolated from the surface of abalone seedlings in Shandong Province (37°48′38.3″ N, 120°45′32.3″ E), China [9]. Genomic DNA of the strain was extracted using a Microbial DNA Extraction Kit (Takara, Tokyo, Japan) according to the manufacturer’s instructions. The genomic sequencing of the strain was performed using Illumina Miseq and PacBio RS II sequencing technologies [24]. The 16S rRNA gene sequence similarities between strain Hao 2018 and closely related strains were calculated using the EzBioCloud server. The 16S rRNA gene sequences of strain Hao 2018 and closely related strains were aligned using the fast secondary-structure aware infernal aligner available in the Ribosomal Database Project, and a phylogenetic tree was constructed using MEGA7 software [43].

### 4.2. Sample Collection and Processing

To collect all publicly available genomes putatively belonging to *P. agarivorans*, the genomes of all strains with more than 98.0% 16S rRNA gene sequence similarities to strain DSM 14585^T^ were retrieved from GenBank. More specifically, the ten genomic sequences of strains *P. agarivorans* DSM 14585^T^, *P. agarivorans* NW 4327, *P. agarivorans* S816, *P. atlantica* ECSMB14104, *P.* sp. P1–11, *P.* sp. P1–30, *P.* sp. TB51, *P. espejiana* DSM 9414^T^, and *P. atlantica* T6c used in this study were obtained from GenBank.

### 4.3. Average Nucleotide Identity (ANI), In Silico DDH, and Pan- and Core-Genome Analyses

To investigate the phylogenetic relationships within strains putatively belonging to *P. agarivorans*, the ANI and in silico DDH values as well as the genome sequence-based phylogenetic relationship comparisons were calculated for strains presumed to belong to *P. agarivorans* and strains belonging to the other *Pseudoalteromonas* species using the stand-alone program [44] and the server-based genome-to-genome distance calculator ver. 2.1 [45]. The general genome features of *P. agarivorans* DSM 14585^T^, *P. agarivorans* Hao 2018, *P. agarivorans* NW 4327, *P. agarivorans* S816, *P. atlantica* ECSMB14104, *P.* sp. P1–11, *P.* sp. P1–30, *P.* sp. TB51, *P. espejiana* DSM 9414^T^, and *P. atlantica* T6c were investigated primarily using the National Center for Biotechnology Information (NCBI) Prokaryotic Genomes Automatic Annotation Pipeline for auto-annotation (Table 1). Pan- and core-genomes were analyzed using the Bacterial Pan Genome Analysis (BPGA) pipeline with a 50% sequence identity cutoff [46]. The expected number of genes was predicted, and the fitted curve was represented by Heaps’ law. The expression formula of Heaps’ law is as follows: *n* = *k* × *N*^−*α*^, where *n* is the size of the pan-genome, *N* is the number of genomes, and *k* and *α* are the constants of the curve [18,47].

### 4.4. Clusters of Orthologous Groups (COG) Analysis

Functional genes from the *P. agarivorans* Hao 2018 strain were classified according to the classification catalog of the COG database in eggNOG-mapper software [48]. eggNOG v4.0 software using the Basic Local Alignment Search Tool (BLAST) with a critical value of 1 × 10^−6^.

### 4.5. Kyoto Encyclopedia of Genes and Genomes (KEGG) Enrichment Analyses

Pathway annotations for KEGG Orthology (KO) and the protein-encoding genes of *P. agarivorans* Hao 2018 strains were mainly performed using the KEGG Automatic Annotation Server [49]. After the KO annotation was completed, mapping of the specific metabolic pathways to the corresponding KEGG pathway was conducted using the iPath v2 module [50].

### 4.6. Transcriptome Sequencing and Data Analyses

Following the general rules of biosynthesis, transcriptome sequencing was carried out by selecting the 2 h time node of the exponential phase and the 24 h time node of the stationary phase for the *P. agarivorans* Hao 2018 strain cultured in a medium containing 45 g/L glucose, 2.5 g/L (NH_4_)_2_SO_4_, and 45 g/L bay salt (pH 7.6–7.8). Fermented cells were harvested by centrifugation at 10,000× *g* for 5 min at 4 °C. Total RNA from the cells was extracted using a TransZol Kit according to the manufacturer’s instructions (TransGen Biotech, Beijing, China). RNA quality was evaluated using NanoDrop spectrophotometer and Agilent 2100 Bioanalyzer. The rRNA was removed by Ribo-Zero rRNA Magnetic Kit, and the mRNA was processed using Truseq TM RNA sample prep Kit. Random primers and SuperScript III reverse transcriptase were used to make the first strand of cDNA, and dUTP instead of dTTP was used to synthesize the second strand. The second strand of cDNA with adaptor junction was digested by uracil-N-glycosylase, and then the library fragments were enriched by PCR and sequenced by Agilent 2100 Bioanalyzer on an Illumina HiSeq 2500 sequencing platform. Mapping of the mRNA sequencing reads against all coding sequences (CDS) of strain Hao 2018 was performed using Burrows-Wheeler Aligner software [51] based on the matching criteria of the best match with a 20 bp minimum alignment and 90% minimum identity. The sequencing process and preliminary analysis were performed by Shanghai Paisano Biotechnology Co (Shanghai, China).

### 4.7. Transcriptome Data Analyses

The Cutadapt (Version 1.2.1) software was used to filter the raw data for reads containing joints and low quality, allowing 20% base mismatches while removing reads with average quality scores below Q20 [52]. The filtered reads were then compared to the reference genome using Bowtie2 [53] (http://bowtie-bio.sourceforge.net/index.shtml, accessed on 5 May 2018) to analyze the distribution of coverage of sequenced reads on the genes. Read count values on each gene were counted by HTSeq 0.6.1p2 [54] (https://htseq.readthedocs.io/en/master/, accessed on 5 May 2018) as raw expression. RPKM (Reads Per Kilobase per Million mapped reads) was used to normalize the expression volume, making the gene expression levels comparable between samples. Expression pattern correlations of genes between samples were assessed and principal component analysis (PCA) was performed using the DESeq2 package [55] based on the expression of each sample. Differentially expressed genes were analyzed by DESeq (Version 1.18.0) package [56] with the screening condition of expression fold difference log2|fold change|>1 and significance *p*-value < 0.05, and the screening results were visualized using the graphical visualization package ggplot2. The calculation of *p*-values for GO terms with significant enrichment (*p* < 0.05) of differential expressed genes for each mapping to the Gene Ontology (http://geneontology.org/ were accessed on 5 May 2018) database using the hypergeometric distribution method in the context of the whole genome of *P. agarivorans* Hao 2018 [57].

## 5. Conclusions

In this study, we analyzed the phylogenetic and genomic diversity of *P. agarivorans*. The pan- and core-genomic analyses found that it had an open genome that evolved to adapt to various environmental changes. The COG, KEGG, and BLAST analysis results show that *P. agarivorans* strains have promising carbohydrate fermentation characteristics. In addition, we screened genes encoding key enzymes that may be related to extracellular polysaccharide synthesis by differentially expressed genes analysis. In summary, our study provided information regarding the phylogenetic characteristics and metabolic fermentation pathways of the *P. agarivorans* strains. Further analysis of the genomic, transcriptomic and metabolic characteristics of *P. agarivorans* Hao 2018 might reveal its carbohydrate fermentation properties and its EPS synthesis pathway, providing a reference for the development of extracellular polysaccharide products with promising applications.

## Figures and Tables

**Table 1:** General features for all the genome sequences used in this study.

Strain Name in GenBank (Accession No.)	Genome Status ^a^ (No. of Contigs)	Total Size (Mb)	G + C Contents (%)	No. of Genes	Completeness (%) ^b^	Contamination Rate (%) ^b^	Isolation Sources and Geographic Location
*P. agarivorans* DSM 14585^T^ (NZ_CP011011–2)	C (2)	4.54	40.87	4119	99.56	0.76	Seawater and Ascidians; Pacific Ocean
*P. agarivorans* Hao 2018 (NZ_CP033065–7)	C (3)	4.56	40.83	4105	99.98	0.40	Surface of abalone seedlings; China: Penglai
*P. agarivorans* NW 4327 (NZ_AZIO00000000)	D (129)	4.48	40.90	4035	99.94	3.91	Sponge; Great Barrier Reef
*P. agarivorans* S816 (NZ_APME00000000)	D (133)	4.42	40.90	4062	100	0.25	Seawater; Sierra Leone: Waterberg
*P. atlantica* ECSMB14104 (NZ_CP023464)	C (1)	3.66	41.00	3331	91.92	0.25	Seawater; East China Sea
*P.* sp. P1–11 (NZ_LJSP00000000)	D (31)	4.38	41.00	3923	100	0.56	Hydractinia echinate; USA: Woods Hole
*P.* sp. P1–30 (NZ_LKBC00000000)	D (35)	4.33	40.90	3869	100	0.13	Hydractinia echinate; USA: Woods Hole
*P.* sp. TB51 (NZ_AUTO00000000)	D (369)	4.63	40.90	4394	99.25	1.01	Marine sponge; Antarctic
*P. espejiana* DSM 9414^T^ (NZ_CP011028–9)	C (2)	4.50	40.30	4051	99.75	0.51	Coastal sea Water; USA: Northern California
*P. atlantica* T6c (NC_008228)	C (1)	5.18	44.60	4319	99.92	0.13	Seawater; Atlantic

^a^ Genome status: D, draft genome sequence; C, complete genome sequence. ^b^ Determined by CheckM.

**Table 2:** Expression of genes related to polysaccharide transport.

Annotation	Locus Tag	2 h_RPKM	24 h_RPKM
glucose/galactose MFS transporter	D9T18_18920	210.32	90.40
MFS transporter	D9T18_05400	150.59	42.61

**Table 3:** Gene expression related to nucleotide sugar synthesis.

Annotation	Locus Tag	2 h_RPKM	24 h_RPKM
UDP-glucuronate-epimerase	D9T18_02060	188.19	45.88
dTDP-4-dehydrorhamnose 3,5-epimerase	D9T18_02110	657.00	66.08
dTDP-4-dehydrorhamnose reductase	D9T18_02115	582.10	45.39
dTDP-glucose 4,6-dehydratase	D9T18_02120	800.04	66.56
UTP--glucose-1-phosphate uridylyltransferase	D9T18_02300	2430.93	533.75
UDP-glucose 6-dehydrogenase	D9T18_02305	838.21	375.42
UTP--glucose-1-phosphateuridylyltransferase	D9T18_06755	324.19	271.59
glucokinase	D9T18_07205	261.87	106.27
phosphoglucomutase	D9T18_08400	476.66	74.74
UTP--glucose-1-phosphate uridylyltransferase	D9T18_11960	678.51	358.89

**Table 4:** Expression of genes related to glycosyltransferase synthesis.

Annotation	Locus Tag	2 h_RPKM	24 h_RPKM
glycosyl transferase	D9T18_02030	156.40	15.57
glycosyl transferase family 2	D9T18_02205	145.28	48.82
rhamnosyltransferase	D9T18_02235	435.05	33.75
glycosyl transferase	D9T18_03335	8.95	37.95
glucosyl transferase family 2	D9T18_06790	166.71	43.81
glucosyl transferase family 2	D9T18_06795	162.09	27.37
glycosyl hydrolase family 16	D9T18_17140	9.01	15.14
glycosyl transferase	D9T18_17235	23.92	15.30
glycosyl transferase family 2	D9T18_19775	150.85	44.41

## Supplementary Materials

The following are available online at https://www.mdpi.com/article/10.3390/md20040248/s1. Figure S1. A neighbor-joining tree based on 16S rRNA gene sequences, showing the phylogenetic relationships between strain Hao 2018 and closely related *Pseudoalteromonas* type strains by MEGA7. Bootstrap values are shown on branching nodes as percentages of 1000 replicates for values over 70%. *Pseudoalteromonas aestuariivivens* DB-2T (KT366926) was used as an outgroup. Bar, 0.002 changes per nucleotide position. Figure S2. RPKM density discretization (**a**) and (**b**) statistical box plots of RPKM density distribution in the two groups of samples. Figure S3. RPKM saturation in two samples of (**a**) 2 h and (**b**) 24 h. Figure S4. Correlation test for two samples of 2 h and 24 h. Figure S5. Principal component analysis. Table S1. Statistics of data filtering results. Table S2. Sequence mapping result statistics.
