# Supplementary material for: Pan-Genomic and Transcriptomic Analyses of Marine Pseudoalteromonas agarivorans Hao 2018 Revealed Its Genomic and Metabolic Features
Source: Mar Drugs. 2022 Mar 31;20(4):248. doi: 10.3390/md20040248 (PMC9027991; doi:10.3390/md20040248)
Supplement: Supplementary file 1 [file marinedrugs-20-00248-s001.zip › marinedrugs-1646545-supplementary.pdf]

Supplementary Information

Pan-genomic and Transcriptomic Analyses of Marine *Pseudoalteromonas agarivorans* Hao 2018 Revealed Its Genomic and Metabolic Features

Yuhao Jv [1], Chenxiang Xi [1], Yanqiu Zhao [1], Wei Wang [1], Yiling Zhang [1], Kai Liu [1], Wenlin Liu [1], Kai Shan [1], Chunlei Wang [1], Ruiwen Cao [1], Cunxi Dai [1], Yiting Jv [1], Wenxing Zhu [1], Haiyong Wang [1], Qiuxia He [1] and Lujiang Hao [1,2,*]

[1] School of Bioengineering, Qilu University of Technology (Shandong Academy of Sciences), Jinan 250353, China; 10431201111@stu.qlu.edu.cn (Y.J.); 1043119587@stu.qlu.edu.cn (C.X.); 10431211120@stu.qlu.edu.cn(Y.Z.); 10431211258@stu.qlu.edu.cn (W.W.); 10431200980@stu.qlu.edu.cn (Y.Z.); kai_liu2018@163.com (K.L.); 1043118323@stu.qlu.edu.cn (W.L.); kai_shan@163.com (K.S.); chunlei_wang79@163.com (C.W.); rwcao@qlu.edu.cn (R.C.); 201996080002@stu.qlu.edu.cn (C.D.); 202181010040@stu.qlu.edu.cn (Y.J.); wenxingzhu@qlu.edu.cn (W.Z.); wangchemical@126.com (H.W.); heqiuxia2022@126.com (Q.H.)

[2] State Key Laboratory of Biobased Material and Green Papermaking, Qilu University of Technology (Shandong Academy of Sciences), Jinan 250353, China.

* Correspondence: lujiang_hao@qlu.edu.cn

Figure S1. A neighbor-joining tree based on 16S rRNA gene sequences, showing the phylogenetic relationships between strain Hao 2018 and closely related *Pseudoalteromonas* type strains by MEGA7. Bootstrap values are shown on branching nodes as percentages of 1000 replicates for values over 70%. *Pseudoalteromonas aestuariivivens* DB-2T (KT366926) was used as an outgroup. Bar, 0.002 changes per nucleotide position.

Figure S2. RPKM density discretization (**a**) and (**b**) statistical box plots of RPKM density distribution in the two groups of samples.

Figure S3. RPKM saturation in two samples of (**a**) 2h and (**b**) 24h.

Figure S4. Correlation test for two samples of 2h and 24h.

Figure S5. Principal component analysis.

Table S1. Statistics of data filtering results.

Table S2. Sequence mapping result statistics.

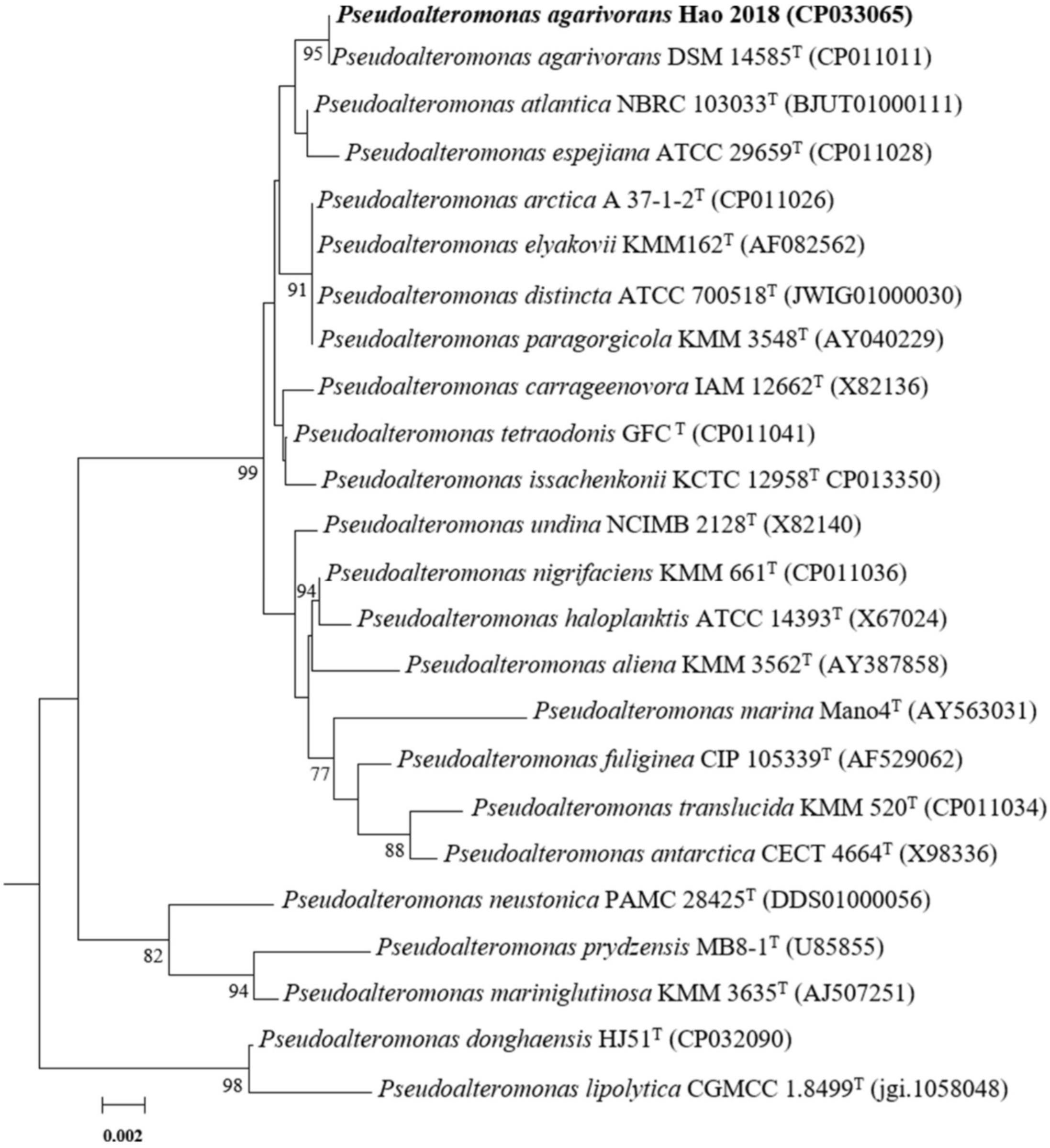

Figure S1. A neighbor-joining tree based on 16S rRNA gene sequences, showing the phylogenetic relationships between strain Hao 2018 and closely related *Pseudoalteromonas* type strains by MEGA7. Bootstrap values are shown on branching nodes as percentages of 1000 replicates for values over 70%. *Pseudoalteromonas aestuariivivens* DB-2T (KT366926) was used as an outgroup. Bar, 0.002 changes per nucleotide position.

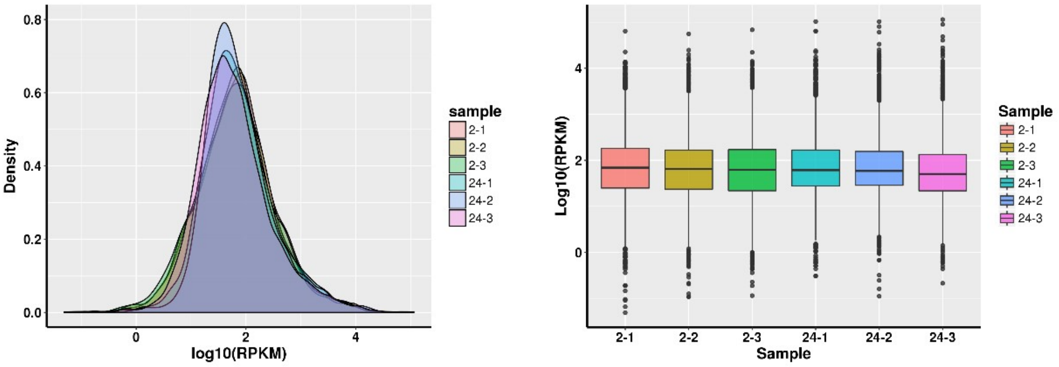

Figure S2. RPKM density discretization (**a**) and (**b**) statistical box plots of RPKM density distribution in the two groups of samples.

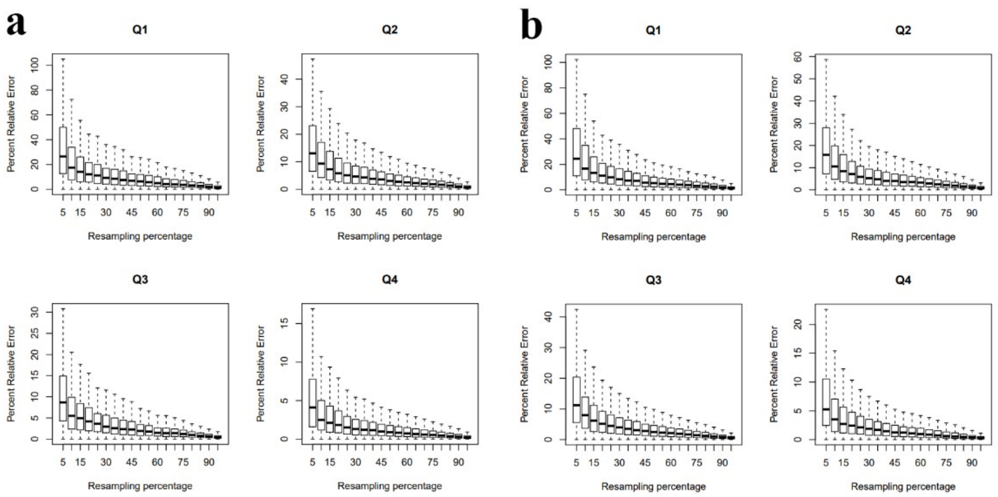

Figure S3. RPKM saturation in two samples of (**a**) 2h and (**b**) 24h.

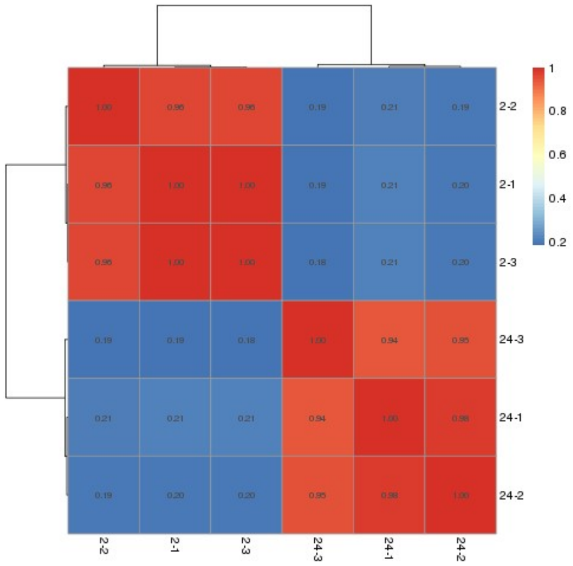

Figure S4. Correlation test for two samples of 2h and 24h.

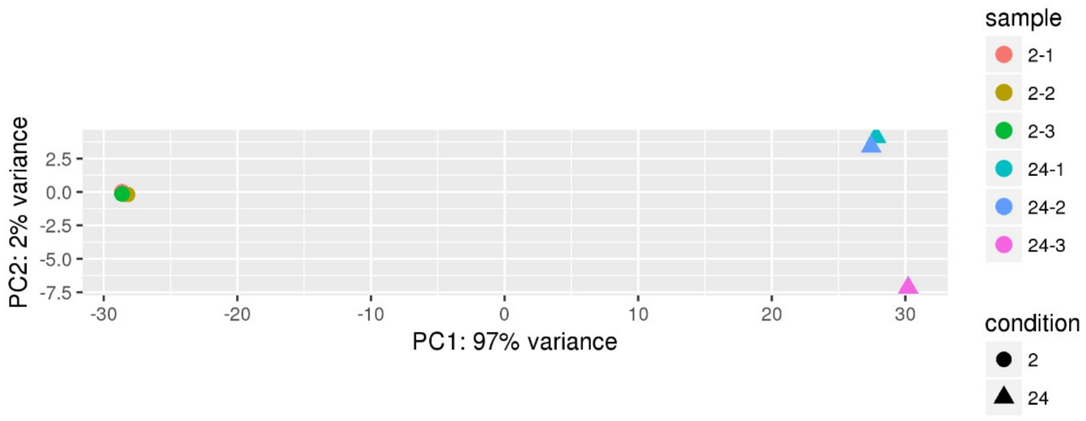

Figure S5. Principal component analysis.

Table S1. Statistics of data filtering results

Sample	Useful Reads	Total Mapped Reads number and percentage		Uniquely Mapped Reads number and percentage		Multiple Mapped Reads number and percentage	
2-1	35,539,354	35,293,023	99.31%	34,100,616	96.62%	1,192,407	3.38%
2-2	32,827,394	32,562,294	99.19%	31,511,500	96.77%	1,050,794	3.23%
2-3	30,147,348	29,885,919	99.13%	28,776,927	96.29%	1,108,992	3.71%
24-1	36,407,882	36,102,399	99.16%	35,573,729	98.54%	528,670	1.46%
24-2	32,234,184	32,034,111	99.38%	31,667,698	98.86%	366,413	1.14%
24-3	36,228,242	35,964,709	99.27%	35,482,252	98.66%	482,457	1.34%

Table S2. Sequence mapping result statistics

Sample	Clean Reads No.	Clean Data (bp)	GC (%)	Clean Reads (%)	Clean Data (%)
2-1	35,539,354	5,315,365,568	42.93	99.74	99.45
2-2	32,827,394	4,911,816,504	43.26	99.72	99.47
2-3	30,147,348	4,510,663,522	43.03	99.71	99.45
24-1	36,407,882	5,448,102,540	44.49	99.36	99.12
24-2	32,234,184	4,824,059,398	43.86	99.70	99.47
24-3	36,228,242	5,419,476,838	44.34	99.69	99.42
